# *Portulaca oleracea* seeds’ extract alleviates acrylamide-induced testicular dysfunction by promoting oxidative status and steroidogenic pathway in rats

**DOI:** 10.1186/s12906-021-03286-2

**Published:** 2021-04-14

**Authors:** Ola M. Farag, Reham M. Abd-Elsalam, Shymaa A. El Badawy, Hanan A. Ogaly, Muhammad A. Alsherbiny, Kawkab A. Ahmed

**Affiliations:** 1 General Organization for Veterinary Services, Giza, Egypt; 2 Department of Pathology, Faculty of Veterinary Medicine, Cairo University, Giza, 12211 Egypt; 3 Department of Pharmacology, Faculty of Veterinary Medicine, Cairo University, Giza, 12211 Egypt; 4 Department of Biochemistry, Faculty of Veterinary Medicine, Cairo University, Giza, 12211 Egypt; 5 Department of Pharmacognosy, Faculty of Pharmacy, Cairo University, Cairo, 12613 Egypt

**Keywords:** Acrylamide, Oxidative stress, Histopathology, Apoptosis, *Portulaca oleracea* seeds, Testosterone, Steroidogenesis

## Abstract

**Background:**

Acrylamide (ACR) is a widespread industrial and food contaminant that garnered considerable attention for its carcinogenic, neurotoxic, and reproductive toxic effects. The antioxidant effects of *Portulaca oleracea* seeds extract (POS) and its fertility-enhancing effects were inspiring to evaluate the protective potential and pinpoint the mechanisms and molecular targets of the UPLC-MS fingerprinted POS extract on ACR-induced testicular toxicity in rats.

**Methods:**

Male Wistar rats were divided into 6 equal groups of negative control, ACR model (10 mg/kg b.wt.), POS at doses of (200 and 400 mg/kg b.wt.) and POS-treated ACR groups. All treatments were given by oral dosing every day for 60 days.

**Results:**

Administration of POS extract reversed the ACR-induced epididymides weight loss with improved semen quality and count, ameliorated the ACR-decreased testicular lesion scoring, testicular oxidative stress, testicular degeneration, Leydig cell apoptosis and the dysregulated PCNA and Caspase-3 expression in a dose-dependent manner. It upregulated the declined level of serum testosterone and the expression of steroidogenic genes such as CYP11A1 and 17β3-HSD with an obvious histologic improvement of the testes with re-establishment of the normal spermatogenic series, Sertoli and Leydig cells.

**Conclusions:**

The supplementation with POS extract may provide a potential protective effect for ACR-induced testicular dysfunction which is mediated by its antioxidant, antiapoptotic and steroidogenic modulatory effects.

**Supplementary Information:**

The online version contains supplementary material available at 10.1186/s12906-021-03286-2.

## Background

The increased exposure to environmental toxicants has been associated with important public health implications. Acrylamide (ACR) is a reactive chemical widely used in several industrial processes, including the production of paper, dyes, soil conditioners, cosmetics, as well as for wastewater and municipal drinking water treatment [1]. In addition to these environmental sources of exposure which could be limited to certain occupations, the presence of a high ACR content in carbohydrate-rich heat-processed foods and cigarette smoke constitutes the key impact in human ACR burden [2].

Given its low molecular weight and high solubility, ACR can readily pass most biological membranes, metabolized by CYP2E1 into a more active epoxide derivative, glycidamide, that contributes to the development of ACR toxic actions [3, 4]. Studies on humans and experimental animals demonstrated that ACR is genotoxic [5], carcinogenic [6], neurotoxic [7], and endocrine disruptor [8]. In the testis, ACR accumulates and disrupt testicular functions and reproductive health [9, 10]. Studies on male rodents suggested that chronic ACR exposure resulted in testicular cytotoxicity indicated by vacuolization, multiple nuclei formation, abnormal giant cell, atrophy of the seminiferous tubules and apoptosis. Moreover, sperm chromosomal aberration, poor sperm viability and count, as well as impaired spermatogenesis were reported [11]. In addition, the ACR-induced reproductive toxicity is associated with a dramatic reduction in testosterone level due to disruption of the Leydig cells steroidogenic pathway [10]. Based on this mechanistic understanding, studies are directed to develop new therapies for ACR and other reproductive toxicants that share its toxic mode of action.

*Portulaca oleracea* L (Purslane or Ma-Chi-Xian) is a widespread invasive weed belonging to the family Portulacaceae. Purslane or “global panacea”, as termed by WHO, has extensive distribution in diverse environments worldwide [12]. Phytochemical analysis of purslane has confirmed its high nutritional value due to its high content in α-linolenic acid, carotenoids, tocopherols and ascorbic acid [13]. Purslane has been used as a vegetable and as a folk medicine for a wide range of pharmacological effects including bronchodilatory, antimicrobial, Antitussive [14, 15], analgesic, anti-ulcerogenic, anti-inflammatory [16], neuroprotective, and anti-diabetic effects [17]. Also, purslane exerted anti-fertility effect in female rats by disrupting ovulation and female fertility hormones [18], while it diminished aging alterations in female reproductive system in aged mice [19]. Interestingly, *Portulaca oleracea* proved to have an aphrodisiac effect in male rats [20, 21]. Furthermore, purslane protected against reproductive toxicity induced by the anti-epileptic drug, carbamazepine as manifested by restored male fertility hormones and cholesterol level in testis. The latter effect may be due to its hormonal and antioxidant effects [22]. However, further research is still required to investigate POS’s various male fertility effects and its underlying cellular and molecular mechanisms.

Remarkably, none of the previous studies measured the protective effects of *Portulaca oleracea* seeds (POS) in alleviating the adverse events associated with ACR-induced reproductive toxicity. Therefore, the present study was designed to determine whether the purslane seeds extract could attenuate ACR-induced alterations in testicular tissue, and explore the possible underlying molecular mechanisms.

## Materials and method

### *Portulaca oleracea* seeds extraction and UPLC-MS fingerprinting

The *P. oleracea* seeds were purchased from a local market of medicinal plants (Haraz, Cairo, Egypt) and were authenticated at the herbarium of Botany Department, Faculty of Science, Cairo University, Giza, Egypt, using the Chinese pharmacopeia [23]. Former extraction protocol was followed with few modifications [24]. Briefly, 250 g of powdered seeds was blended with 70% ethanol in a sealed glass vessel. Then the mixture was allowed to settle down for 72 h, in the dark at room temperature, and then was filtrated. After completion of maceration (3 times), the solvent was evaporated under vacuum using a rotary evaporator (Heidolph, Laborta 4000) at a temperature below 50 °C and RPM 60 to yield a viscous extract. The obtained extract was stored at − 20 °C until used. The yielded extract weight was 36.8 g (extraction yield: 25.5% w/w). For the preparation of doses, the extract was freshly dissolved in distilled water using a 2% tween 80. We conducted UPLC-MS fingerprinting of the used POS extract in ACQUITY ultra-performance liquid chromatography (UPLC) (Waters Corp., USA) coupled with SYNAPT G2-S (Waters Corp., USA) mass spectrophotometer and results were previously reported [25].

### Chemicals and reagents

All chemicals used in the study were of analytical or HPLC grade. Acrylamide (CAS number 79–06-1) was purchased from Sigma Aldrich. Kits for measuring oxidative stress parameters MDA (MD 25 29), GSH (GR 25 11) and SOD (SD 25 21) were purchased from Biodiagnostic Co. (Dokki, Giza, Egypt). In addition to, testosterone ELISA kit (Abcam 108,666, Cambridge, UK).

### Animals and experimental protocol

A total number of 30 male adult Wistar rats weighing 190–220 g, 2 months of age were obtained from the Animal House Colony at National Organization for Drug Control and Research (NODCR, Egypt). Rats were kept at a constant temperature of (25 ± 1) °C, with a 12 h light/dark cycle with free access to standard rodent chow and water ad libitum throughout the experimental period. After a one-week acclimatization period, rats were randomly assigned to 6 groups. Five rats were allocated per group. The 1st group (normal control): rats received a tween 80 (2%) in distilled water. The 2nd and 3rd groups: rats received POS extract at doses of 200 mg/kg.b.wt., and 400 mg/kg.b.wt., respectively [19]. The 4th group: rats received ACR at a daily dose of 10 mg/kg b.wt. for 60 days [26]. The 5th and 6th groups: rats received POS extract at the former two doses concurrently with ACR. Rats in all groups received the treatments orally 1 ml/rat daily for 60 days [26].

### Sampling

After completion of the experimental period, all animals were anaesthetized under intraperitoneal injection with a mixture of ketamine (90 mg/kg of b.wt.) and xylazine (10 mg/kg b.wt.). Blood samples were collected from the retro-orbital sinuses and serum was harvested, aliquoted and stored at − 80 °C until analysed within 1 month for testosterone concentrations. Then, rats were euthanized by decapitation between 09:00 AM and 11:00 AM to eliminate possible effects due to diurnal variation. Testes and epididymides were immediately removed; weighed and relative weights were calculated individually as testes or epididymides weight/body weight. Testes were allocated into duplicate; where one set was quickly stored at − 80 °C to be used for antioxidant assays and PCR, while the other set was kept in 10% formalin to be used for histopathological examination and immunohistochemistry.

### Sperm analysis

Sperm analysis was guided by the method described in the literature [27]. Briefly, one cauda epididymis of each animal was minced with a scalpel, allowing sperm to be dispersed in 3 mmol/l of Hanks’ balanced salt solution (HBSS) at 37 °C. The suspension was examined for evaluation of motility and viability followed by sperm concentration and morphology. Sperm motility was evaluated immediately by placing a drop of sperm suspension in a pre-warmed (37 °C) microscope slide. The total motility was calculated as the average percentage of motile sperms (progressive plus non-progressive) in the 10 random fields under 100X magnification. Sperm viability was estimated using dye exclusion staining technique (Eosin/Nigrosin) [28], where the red eosin stain penetrates spermatozoa with the damaged cell membrane. Thus, stained spermatozoa were recognized as non-viable, whereas unstained spermatozoa were recognized as viable. Sperm count was assessed by diluting sperm in HBSS by a factor of 1:50. This solution was used to fill the two grids of a Neubauer counting chamber using a micro pipette. Sperms in the four large corner squares were counted under (400X magnification) on an optical microscope. Sperm morphology was evaluated on eosin-nigrosin stained slides under an oil immersion field (1000X magnification) [29]. Sperm cells with hook-shaped heads and no visible defects were considered normal, while those with head or tail defects were considered abnormal and were classified as tail defects, deformed head and detached head.

### Assessment of testosterone hormone

Serum total testosterone was assayed using an ELISA kit following the manufacturer’s instructions (Abcam 108,666, Cambridge, UK).

### Assessment of testicular oxidative stress markers

Tissue homogenates were prepared from frozen testes samples in 0.1 M Tris–EDTA buffer (pH 7.4). The supernatant fractions were used for the spectrophotometric determination of reduced glutathione (GSH), malondialdehyde (MDA), the indicator of lipid peroxidation (LPO), and superoxide dismutase activity (SOD), were performed as reported in the literature [30].

### Histopathological examination of testes

The testes were harvested from different groups then fixed in 10% neutral buffered formalin for 24–48 h. The specimens were processed for obtaining 4 μm paraffin embedding sections then stained with hematoxylin and eosin stain (H&E) [31]. The testicular scoring system was evaluated according to criteria reported in an earlier study [32]. These criteria depend on the scoring of the main spermatogenic cells (spermatogonial cells, primary spermatocytes, secondary spermatocytes and spermatid cells) and Sertoli cells. The score ranged from 10 to 1 in thirty randomly chosen seminiferous tubules in each group under power field X200.

### Immunohistochemical analysis of proliferative cell nuclear antigen (PCNA) and Caspase-3 in testicular tissue

The immune-histochemical analysis of PCNA and Caspase-3 expressions in testicular tissues was performed [33]. In brief, the tissue sections were deparaffinised and rehydrated. The antigenic retrieval was done by pre-treating the tissue specimens with citrate buffer PH 6 for 20 min. The tissue specimens were incubated overnight with one of rabbit polyoclonal anti- PCNA antibody (ab18197; Abcam, Cambridge, UK) with dilution 1:4000 and rabbit anti-Caspase-3 polyclonal antibody (ab13847; Abcam, Cambridge, UK) at 1:50 dilution in a humidified chamber. The tissue sections were thoroughly washed with Tris-buffered saline. The non-specific background was blocked by using a blocking solution. The tissue specimens were incubated with secondary (HRP) antibody (ab205718; Abcam, Cambridge, UK). The tissue sections were washed three times with Tris-buffered saline. The tissue reaction was visualized by using DAB (Sigma) as a chromogen. The tissue sections were washed three times with Tris-buffered saline then counterstained with Mayer hematoxylin and mounted. The images were analysed by Image J Analyser. The colour density of the immune-positive cells was evaluated in random five fields/ section. In each group, five sections were evaluated.

### Quantitative mRNA expression analysis by real-time PCR (qRT-PCR)

The total cellular RNA was purified from testes using Trizol reagent following the instructions provided (Invitrogen, Carlsbad, CA). The concentration and purity of the isolated RNA were evaluated by 260/280 nm UV absorbance ratios. The first-strand cDNA was synthesized by reverse transcription using oligo-(dT) primer and M-MLV first chain synthesis kit according to the manufacturer’s protocol (Invitrogen). The expression level of testicular cytochrome P450, family 11, subfamily a, polypeptide 1 (CYP11A1) and hydroxysteroid 17-beta dehydrogenase 3 (17β3-HSD) genes were analysed in a Real-Time PCR System (Applied Biosystems, U.S.A.) using SYBR Green Mix (Invitrogen) and the following primers for CYP11A1, forward: 5′- AGAAGCTGGGCAACATGGAGTCAG-3′, reverse: 5′-TCACATCCCAGGCAGCTGCATGGT-3′ (NM_017286.3); 17β3-HSD, forward: 5′- TTTCTTCGGGAGTAGGGGTTC-3′, reverse: 5′-TCATCGGCGGTCTTGGTCG-3′ (NM_054007.1); and β actin, forward: 5′-ATGGTGGGTATGGGTCAG-3′, reverse: 5′- CAATGCCGTGTTCAATGG-3′ (NM_031144.3). The cycling condition was as follows: 95 °C for 5 min, followed by 40 cycles of 95 °C for 10 s, and 56 °C and 50 °C for 30 s for CYP11A1 and 17β3-HSD, respectively. The relative gene expression was normalized to β-Actin. Each assay was conducted in 3 replicates, and the fold changes of each target gene per average of β actin were calculated according to Comparative Ct (2^−ΔΔCt^) method, with a value of 1.0 used as the control [34].

### Statistical analysis

All experiments were performed in triplicates. The data were expressed as mean ± standard deviation (SD) or standard error (SE) where *n* = 5. Statistical analyses were carried out using SPSS software package (version 24.0.) and performed using one-way ANOVA followed by L.S.D. test and Duncan posthoc test. Values with the different superscript letters e.g. ^‘a, b, c, d, ab, bc’^ are significantly different compared to ACR-treated at *p* < 0.05. All numerical biological and histological results were Pareto-scaled using MetaboAnalyst 5.0 [35] for subsequent multivariate data analysis, correlation and clustering analysis. Both unsupervised Principal Component Analysis (PCA) and supervised Partial Least Squares-Discriminant Analysis (PLS-DA) were explored. In addition, the univariate correlation analysis implementing Pearson R correlations and hierarchical clustering using Euclidean distance measure and Ward clustering algorithm was performed. In addition to random forest supervised learning analysis to rank important discriminatory features according to the mean decrease accuracy where the out-of-bag error (OOB) was 0.033.

## Results

### Effect of POS on relative weights and sperm parameters

**Fig. 1:**
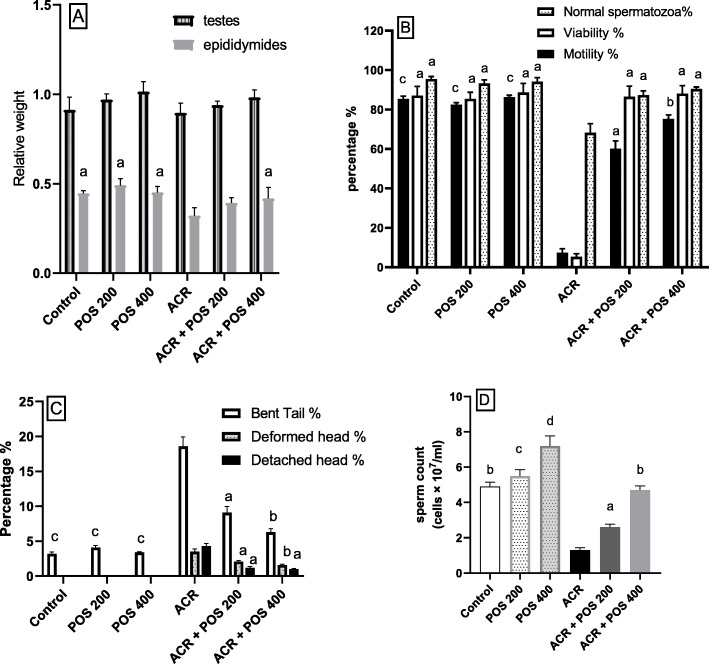
The Effect of *Portulaca oleracea* seeds (POS) extract on (**a**) Relative testicular and epididymides weight, (**b**) Percentage motility, viability and normal spermatozoa, (**c**) Percentage of abnormal spermatozoa (bent tail, deformed head and detached head), (**d**) Sperm count (cells × 10^7^/ml) in male rats with and without acrylamide (ACR)-induced toxicity; 10 mg/kg for 60 days. Values (Mean ± SD, *n* = 5), ‘^a, b, c, d’^ statistically significant compared to ACR group using Duncan post hoc test (*p* < 0.05)

Though exposure to ACR (10 mg/kg) for 60 days significantly diminished the relative epididymides weight, the decrease in relative testes weight was not significant. The concurrent administrations of POS hydro-ethanolic extract (400 mg/kg b.wt.) notably prevented the later toxic effect (Fig. 1a). Sperm parameters including motility, viability, normal spermatozoa percentage and sperm count showed a significant reduction in the ACR group compared to control and non-intoxicated POS-treated groups. Interestingly, no differences were found between the control rats and POS-treated intoxicated rats indicating a significant protective effect of POS against ACR toxicity (Fig. 1b, d). The percentage of abnormal sperms was increased by ACR intoxication; however, sperm morphology improvement and percentage of abnormal sperms reduction were noticed upon concurrent consumption of the POS extract in a dose-related manner (Fig. 1c).

### Effect of POS on the level of testosterone hormones

**Fig. 2:**
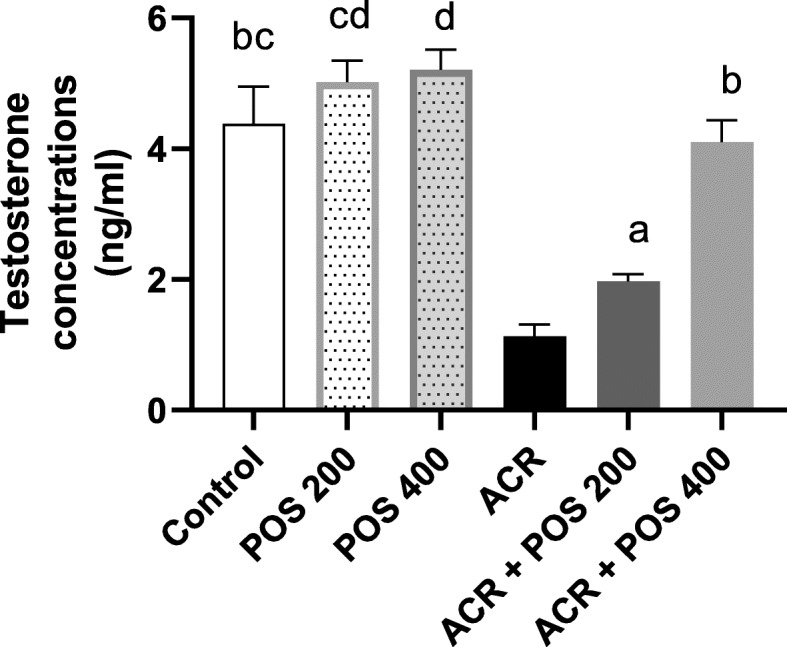
The Effect of *Portulaca oleracea* seeds (POS) extract on testosterone concentration (ng/ml) in male rats with and without acrylamide (ACR)-induced toxicity. Values (Mean ± SD, *n* = 5), ‘^a, b, bc, d, cd’^ statistically significant compared to ACR group using Duncan post hoc test (*p* < 0.05)

Induced acrylamide toxicity in rats over 2 months decreased the testosterone concentrations significantly (*p* < 0.05) compared with the control group. Moreover, the testosterone level in ACR intoxicated male rats was restored by the concurrent treatment with POS extracts in a dose-dependent manner (Fig. 2).

### Effect of POS on testicular oxidative stress markers

Testicular SOD and reduced GSH antioxidant activities were significantly (*p* < 0.05) declined in the ACR group compared to control and POS treated groups. However, elevated LPO exhibited by increased significant MDA (*p* < 0.05) were reported in the ACR group compared with control and POS-treated groups where POS extract reversed the ACR-induced dysregulated oxidative stress markers in a dose-dependent manner (Table 1).

**Table 1:** Effect of POS extract on Testicular oxidant/antioxidant in acrylamide-intoxicated rats

Group	SOD	reduced GSH	Lipid peroxidase (Malondialdehyde)
	U/mg	mg/g	nmol/g
Control	13.3 ± 2.12 ^a^	51.6 ± 4.30 ^a^	31.2 ± 2.42 ^a^
POS 200	12.7 ± 1.99 ^a^	48.3 ± 4.73 ^a^	30.4 ± 3.51 ^a^
POS 400	14.4 ± 1.07 ^a^	54.1 ± 2.82 ^ab^	33.1 ± 2.36 ^a^
ACR	8.5 ± 0.96	36.1 ± 2.91	44.2 ± 3.61
ACR + POS 200	11.4 ± 1.85 ^a^	47.2 ± 4.73 ^a^	32.1 ± 2.26 ^a^
ACR + POS 400	12.8 ± 1.36 ^a^	53.6 ± 5.32 ^ab^	34.5 ± 2.17 ^a^

Values (Mean ± SD, *n* = 5), ‘^a, b, ab’^ statistically significant compared to ACR group using Duncan post hoc test (*p* < 0.05).

### Effect of POS on histopathology of the testes and testicular lesion scoring

**Fig. 3:**
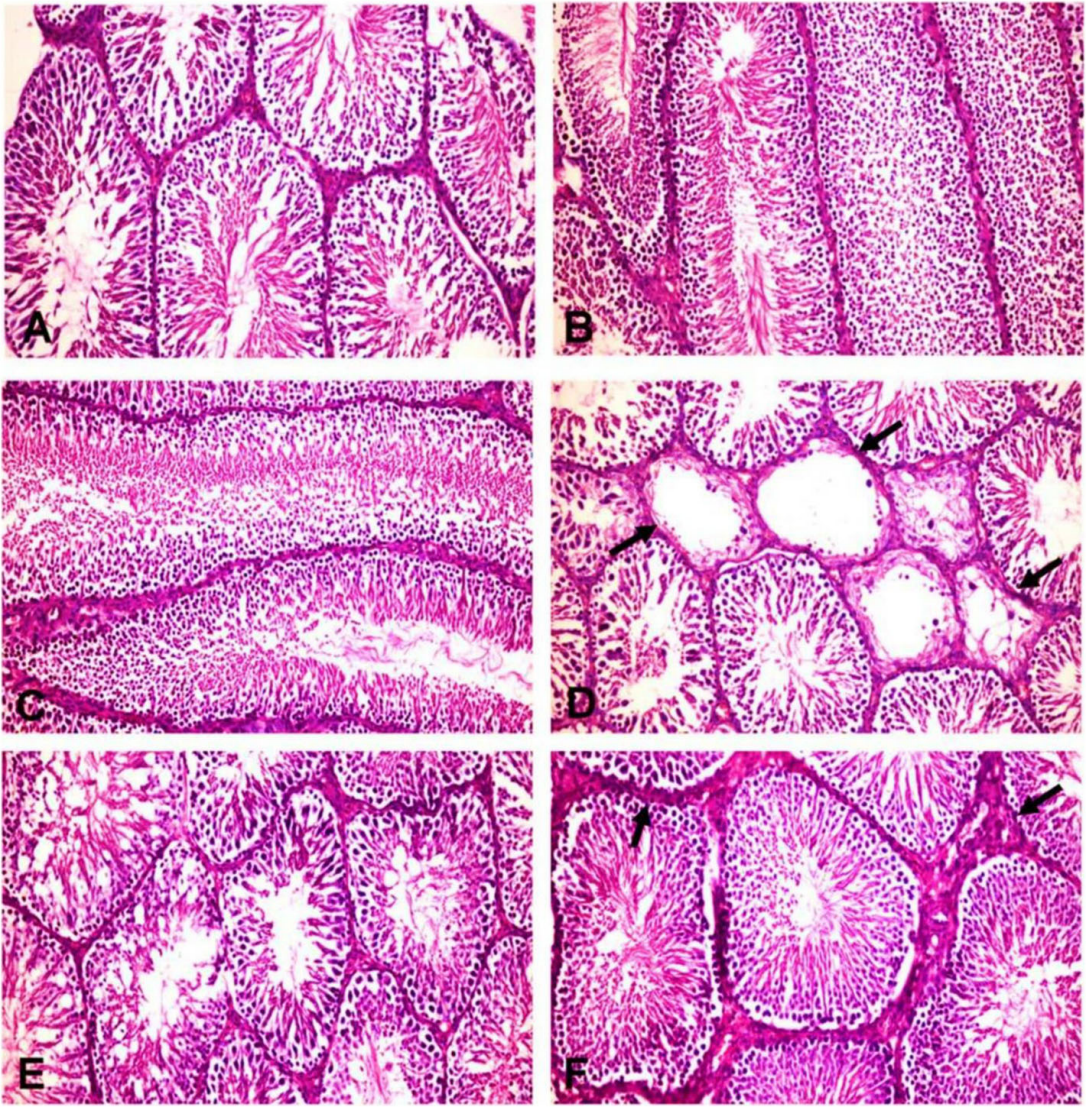
Histopathological pictures of the testes in different experimental groups (H&E X200). **a** Control group, **b** POS (200 mg/kg) and (**c**) POS (400 mg/kg) treated groups; showing normal histology of the seminiferous tubules with main spermatogenic series, Sertoli cells and Leydig cells. **d** ACR treated group showing some seminiferous tubules suffering from marked testicular degeneration (arrows) with a complete absence of spermatogenic series. **e** ACR + POS (200 mg/kg) treated group showing normal seminiferous tubules with incomplete spermatogenic series. **f** ACR + POS (400 mg/kg) treated group showing normal seminiferous tubules with thickening of intertubular tissue

**Fig. 4:**
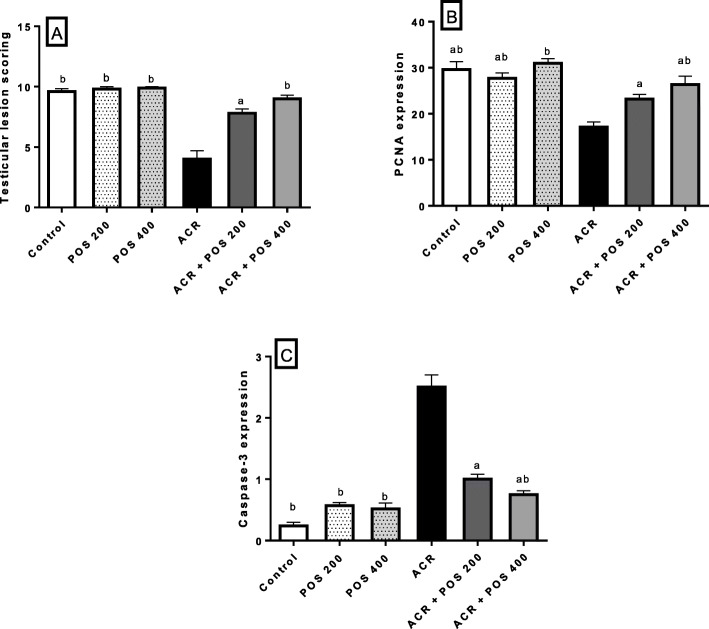
Effects of *Portulaca oleracea* seeds (POS) extract on (**a**) Testicular lesion scorning, (**b**) PCNA expression (**c**) Caspase-3 expression. Values (Mean ± SD, *n* = 5), ^‘a, b, ab’^ statistically significant compared to ACR group using Duncan post hoc test (*p* < 0.05)

Mature normal active seminiferous tubules with normal main spermatogenic, Sertoli and Leydig cells were noticed in the testes of control and POS groups (Fig. 3a, b, c). The ACR group showed severe testicular degeneration in the form of a reduction in the number of the spermatogonial cells, primary and secondary spermatocytes with a marked surge in the number of multinucleate spermatid giant cells (Fig. 3d). Vacuolation of Sertoli cells and apoptosis of Leydig cells were also observed. Several seminiferous tubules contained only vacuolated Sertoli cells with severe loss of germ cells. The epididymal ducts revealed vacuolation and degeneration of their epithelial lining and contained few numbers of spermatozoa. The groups treated with ACR + POS (200 mg/kg) showed a moderate improvement of testicular lesions with incomplete spermatogenic series (Fig. 3e). Whilst ACR + POS (400 mg/kg) revealed obvious enhancement with re-establishment of the normal spermatogenic series, Sertoli and Leydig cells (Fig. 3f). Furthermore, the testicular lesion scoring in the different experimental groups was evaluated (Fig. 4a), where the POS extract restored the ACR-decreased testicular lesion scoring in a concentration-dependent manner.

### Effect of POS on immune-histochemistry of PCNA and Caspase-3

**Fig. 5:**
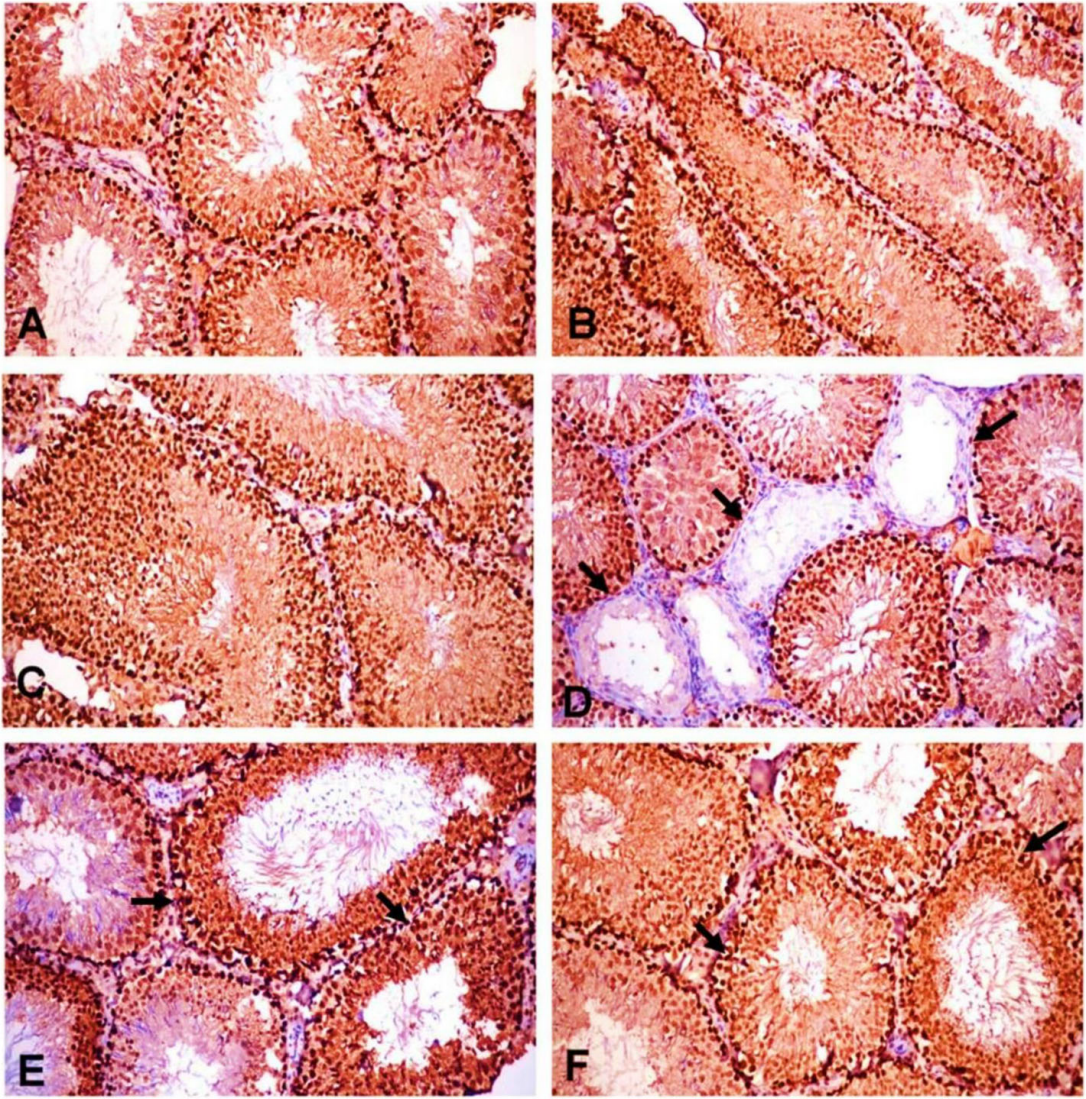
PCNA expression in testes of the different experimental groups (H&E X200). **a** Control group, (**b**) POS (200 mg/kg) and (**c**) POS (400 mg/kg) treated groups; showing strong immunopositive reaction in spermatogenic series. **d** ACR treated group showing very weak immune-positive reaction in some seminiferous tubules (arrows). **e** ACR + POS (200 mg/kg) and (**f**) ACR + POS (400 mg/kg) treated groups showing strong immunopositive reaction in spermatogenic series (arrows) and interstitial cells

**Fig. 6:**
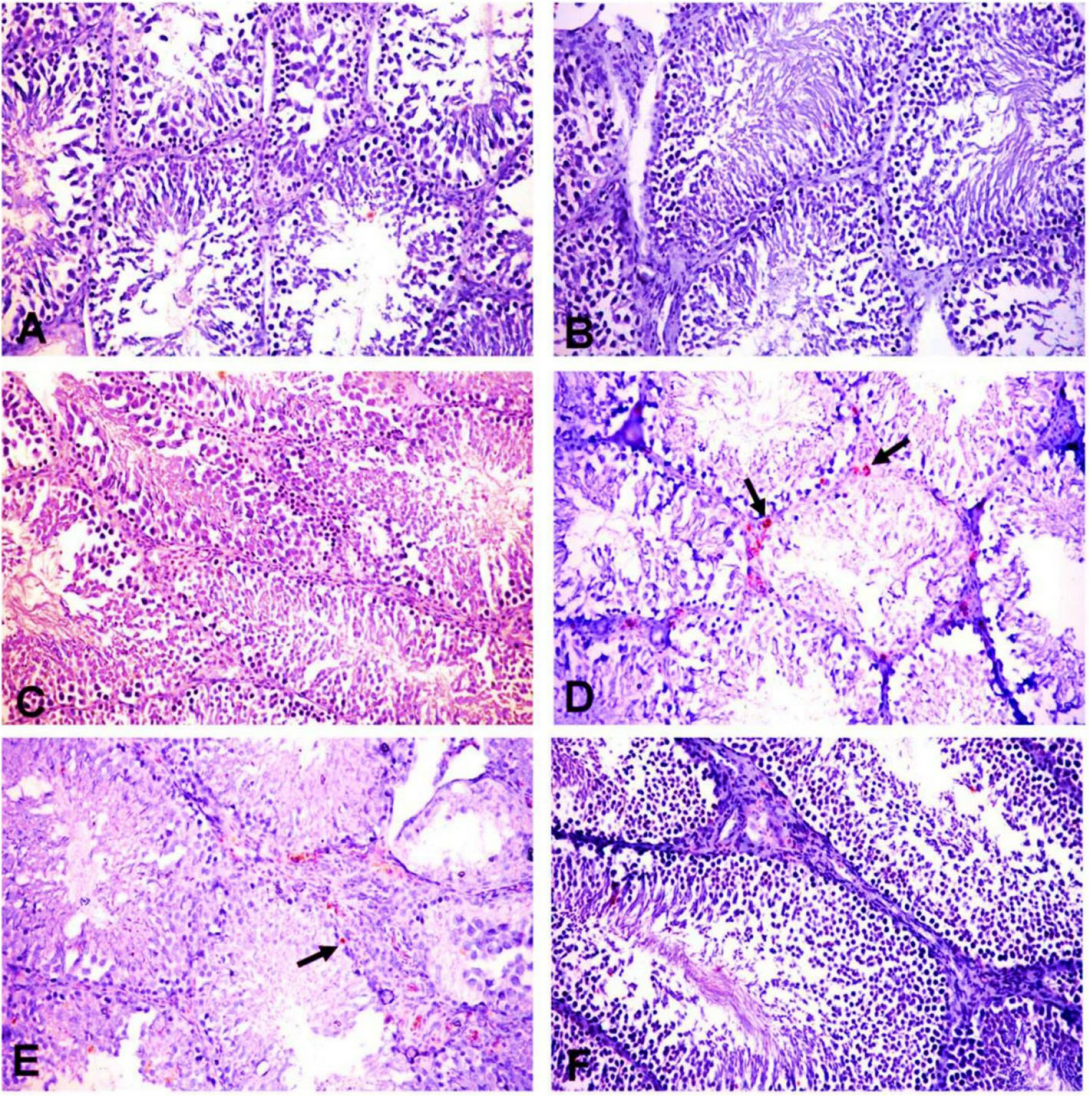
Caspase-3 expression in testes of different experimental groups (H&E X200). **a** Control group, (**b**) POS (200 mg/kg) and (**c**) POS (400 mg/kg) treated groups; showing very weak immunopositive reaction in spermatogenic series and Leydig cells. **d** ACR treated group showing strong immune-positive reaction in Leydig cells (arrows). **e** ACR + POS (200 mg/kg) and (**f**) ACR + POS (400 mg/kg) treated groups showing weak immunopositive reaction in Leydig cells (arrows)

The ACR-induced dysregulations in the PCNA and Caspase-3 expressions in the male rat testes were significantly alleviated by POS extract in a dose-proportional manner (Fig. 4b, c). Likewise, the PCNA expression showed strong expression in both spermatogonial cells and primary spermatocytes, unlike the secondary spermatocytes and spermatid where control, POS-treated groups showed a strong immunopositive reaction (Fig. 5a, b, c). The ACR-intoxication triggered a significant reduction of the PCNA expression in a spermatogenic series (Fig. 5d) compared with the control group. The POS-treated ACR groups revealed a significant increase in the PCNA expression in spermatogenic cells when compared with the ACR group (Fig. 5e, f) with no significant difference could have been observed between the control and POS-treated ACR group in high dose (Fig. 4b). Caspase-3 expression was evaluated in the Leydig cells of the different experimental groups to detect their apoptosis. The control, POS treated groups showed a weak immunopositive reaction in Leydig cells (Fig. 6a, b, c). The ACR group revealed a significant elevation of Caspase-3 expression in Leydig cells (Fig. 6d) in comparison to the control group. The POS-treated ACR groups revealed a significant reduction in immunopositive reaction in Leydig cells compared with the ACR group (Fig. 6e, f).

### Effect of POS on gene expression

**Fig. 7:**
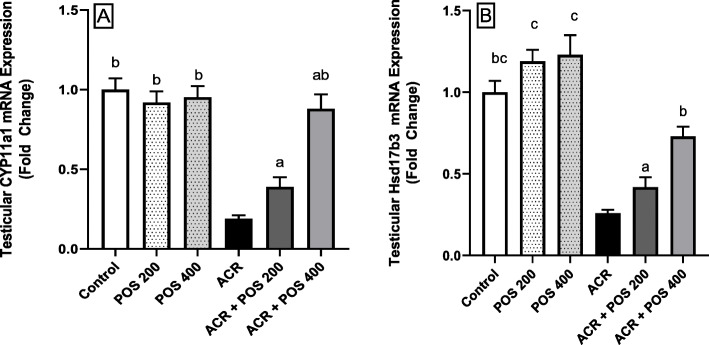
Effects of *Portulaca oleracea* seeds (POS) extract on relative mRNA expression level (fold change) of (**a**) CYP11A1, and (**b**) 17β3-HSD genes in male rats with and without acrylamide (ACR)-induced toxicity using qPCR. Values (Mean ± SD, *n* = 5), ‘^a, b, ab, c, bc’^ statistically significant compared to ACR group using Duncan post hoc test (*p* < 0.05)

The effects of acrylamide exposure on mRNA expression of genes involved in steroidogenesis in male rats were determined (Fig. 7). CYP11A1 is a rate-limiting steroidogenic enzyme that catalyses side-chain cleavage of cholesterol to form pregnenolone. Compared to the control group, mRNA expression of CYP11A1 was significantly decreased by 71% in the acrylamide intoxicated group. Groups treated with POS (400 mg/kg) and (200 mg/kg) showed significant increases in CYP11A1 expression to approximately 88, and 39% of the control, respectively (Fig. 7a). Similarly, gene expression of 17β3-HSD enzyme that plays a significant role in steroidogenesis was also notably reduced in the acrylamide-challenged group to 26% of the control group. Administration of POS in high and low doses restored 17β3-HSD mRNA expression to 73, and 42% of the control, respectively (Fig. 7b). Moreover, non-significant differences between the control and-the POS treated groups were detected for CYP11A1 contrasting the significant tiny incline in the 17β3-HSD mRNA expression (Fig. 7a & b).

### Multivariate, correlation and clustering analyses

**Fig. 8:**
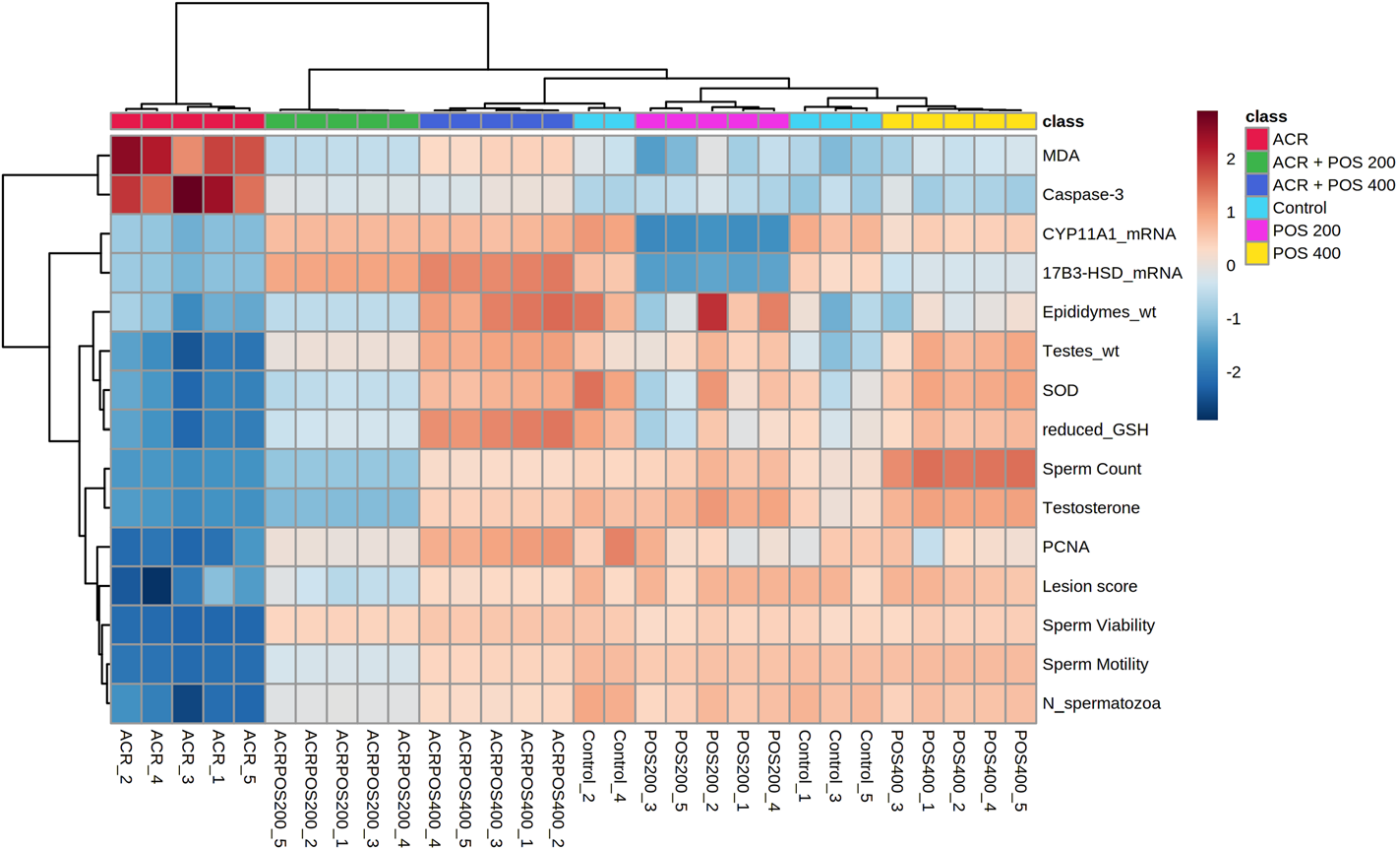
Heat-map summarizes the effect of *Portulaca oleracea* seed extract (POS; 200 or 400 mg/kg) on the studied biomarkers of rats intoxicated with acrylamide (ACR). 17β3-HSD; hydroxysteroid 17-beta dehydrogenase 3, CYP11A1; cytochrome P450, family 11 subfamily a polypeptide 1, GSH; Glutathione, MDA; Malondialdehyde, N_spermatozoa; normal spermatozoa percentage, PCNA; Caspase-3 and proliferating cell nuclear antigen, SOD; superoxide dismutase wt; weight. Coloured boxes indicate the induced effect either upregulation (red) or downregulation (blue)

**Fig. 9:**
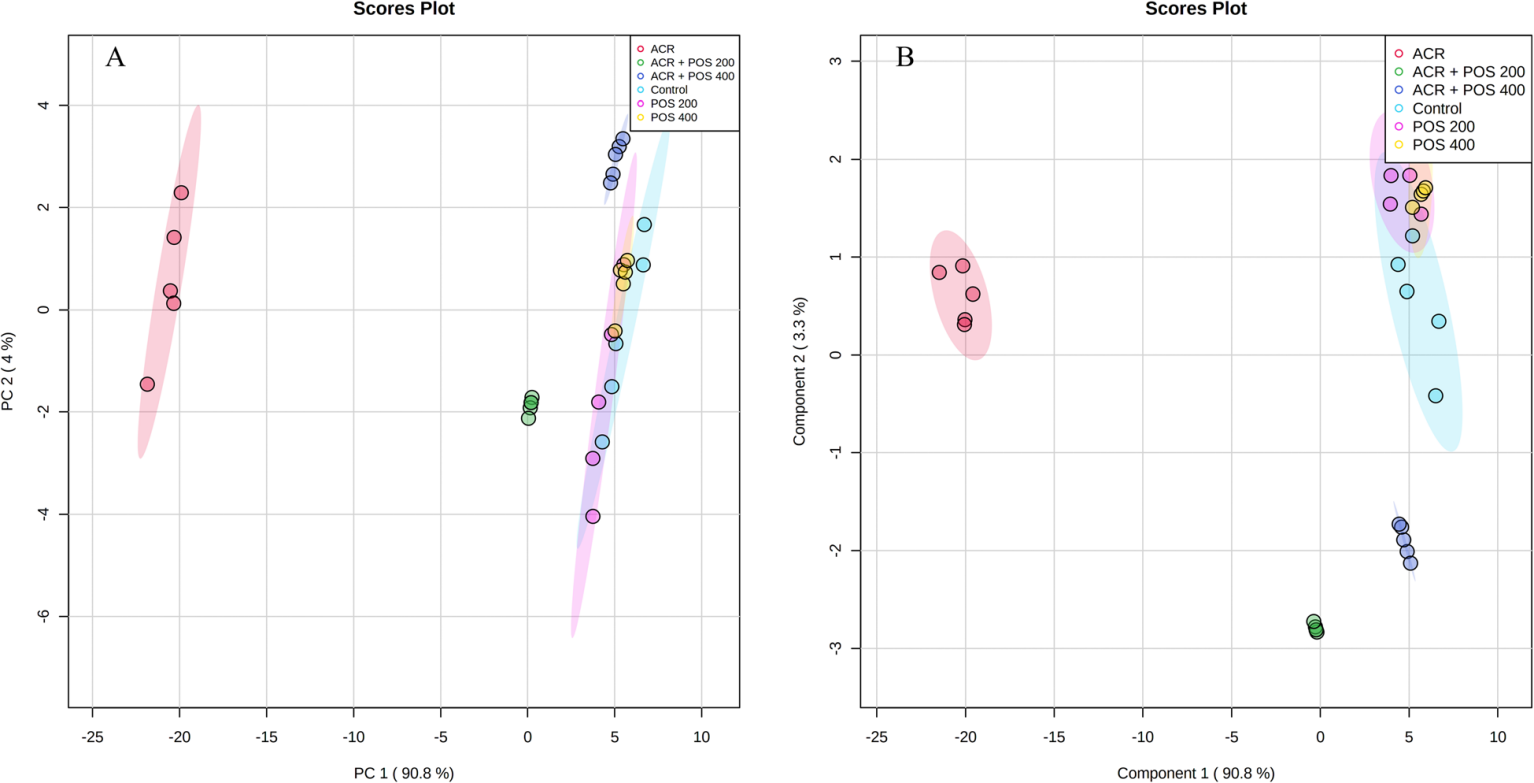
Score plots of (**a**) the Principal component analysis and (**b**) Partial least square discriminant analysis between the selected components of the biomarkers explored during the study of *Portulaca oleracea* seed extract effects (POS; 200 or 400 mg/kg) on the rats intoxicated with acrylamide (ACR)

**Fig. 10:**
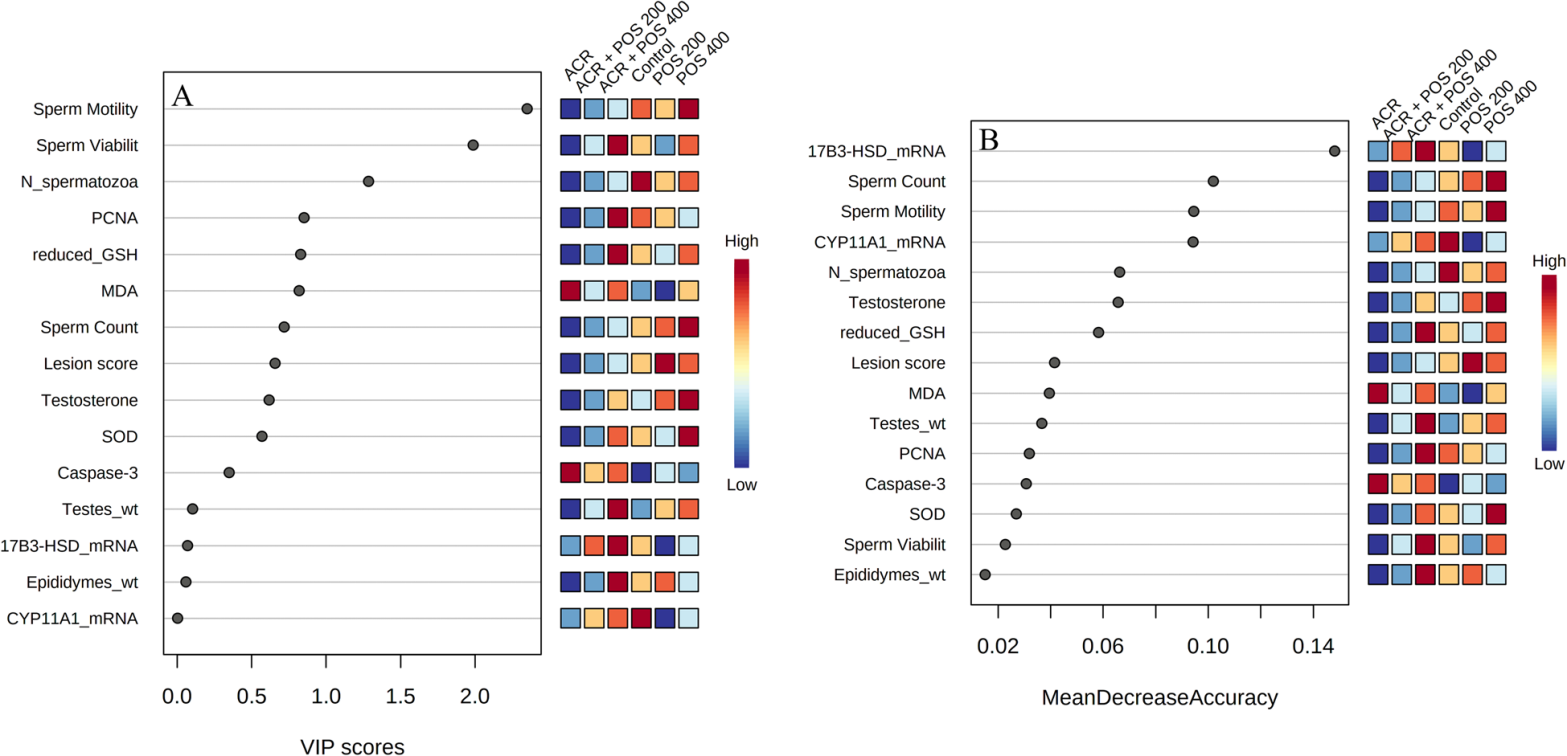
**a** Variable Importance Projection (VIP) score plot detecting the PLSDA identified important biomarkers (**b**) Significant features identified by random forest, ranked by the mean decrease in the classification accuracy during the study of the effect of Portulaca oleracea seed extract (POS; 200 or 400 mg/kg) on acrylamide (ACR) intoxicated rat model. *17β3-HSD*; hydroxysteroid 17-beta dehydrogenase 3, *CYP11A1*; cytochrome P450, family 11 subfamily a polypeptide 1, *GSH*; Glutathione, *MDA*; Malondialdehyde, *N_spermatozoa*; normal spermatozoa percentage, *PCNA*; Caspase-3 and proliferating cell nuclear antigen, *SOD*; superoxide dismutase, *wt*; weight. The coloured boxes on the right indicate the relative regulation of the corresponding marker in each experimental group

Hierarchical cluster analysis and both supervised PCA and unsupervised PLS-DA analyses indicated a well separated ACR model group from others (Figs. 8, 9 and S1). Briefly, the ACR model group was well clustered away from the negative control group while the latter was closely related to the unchallenged groups that received either 200 or 400 mg/kg POS. This confirmed the ACR animal model induction was successful, whereas the unchallenged groups that received POS were overall closely clustered to the negative control away from the ACR model. Interestingly, the ACR-challenged group received 400 mg/kg POS expressed a close relation to the control group indicating the potential effect of this dose to regulate the ACR-disturbed biomarkers under study. Furthermore, the ACR-challenged group received 200 mg/kg was also clustered away from the ACR-model group indicates its potential therapeutic effects, but a weaker effect compared to the high POS dose could have been anticipated augmenting the potentiality of POS in a dose-dependent fashion. All studied biomarkers were furtherly ranked by Variable Importance Projection (VIP) scores in the constructed PLS-DA model and mean decrease accuracy in Random Forest analyses to fetch the important features classifying different experimental groups under study (Fig. 10). Additionally, Pearson r correlation analysis was conducted to visualize the overall correlation between different studied biomarkers to indicate both positively and negatively correlated biomarkers (Fig. S2).

## Discussion

The testicular androgenesis disruption alongside with qualitative and quantitative sperm deterioration via the inclined testicular oxidative stress and apoptosis was revealed by our study as the core ACR-induced reproductive toxicities in male rats. Interestingly, the current investigation reported, for the first time, the protective potential of POS hydro-alcoholic extract against ACR-induced testicular dysfunction in adult male Wistar rats. To the best of our knowledge, the role of POS in the regulation of ACR-induced testicular toxicity has not been investigated. Briefly, the long term ACR exposure (10 mg/kg for 60 days) impairs spermatogenesis as observed by reduction of epididymal sperm count and quality, including deceased sperm viability, motility, normal sperms percentage with inclined sperm morphological abnormalities primarily the tail and head deformities (Fig. 1c) as concurred with previous ACR toxicity studies [36]. These impairments resulted from the ACR-induced degeneration of epithelial cells of the seminiferous tubules in addition to the interfering effect of ACR on kinesin motor protein in the sperm flagella of [37].

No significant difference among the relative testis weights between the experimental groups was observed. However, the relative epididymis weight was significantly reduced in ACR-exposed rats (Fig. 1a). Inconsistent findings regarding the relative testis weights following ACR administration were reported in the literature [38]. The reduction in the epididymis weights may reflect a reduction in the fluid of seminiferous tubules [39] or an inadequate supply of androgen [40].

Accumulating data have proposed oxidative stress as the main mechanism explaining ACR-induced toxicity. ACR and its epoxide metabolites, glycidamide, tend to form covalent adducts with DNA inducing DNA damage, mutations and carcinogenicity. Similarly, ACR and glycidamide can form adducts with thiol groups of most biomolecules leading to depletion of the intracellular thiols that trigger the LPO [41]. ACR specifically interacts with GSH forming glutathione S-conjugates and initiating intracellular electrophiles metabolism [42]. In this sense, the current study showed that ACR administration induces a significant reduction in the testicular SOD enzyme activity with depletion of testicular GSH. Additionally, a significant elevation of LPO product compared to the control group (Table 1). These results coincide with the previous studies [43].

The pro-oxidant/antioxidant imbalance in the testes following ACR intoxication was accompanied by histopathological alterations represented by congestion and interstitial edema, necrosis, calcification and degeneration of spermatogenic cells in the seminiferous tubules with the formation of spermatid giant cells high numbers of apoptotic cells in the seminiferous tubules (Fig. 3). Similar observations of histopathological lesions such as the formation of multinucleated giant cells and production of the rat were reported as well [44].

As the dynamics of testicular germ cells are precisely controlled by a balance between cell proliferation and apoptosis [45]. Therefore, the effects of ACR on testicular apoptotic and proliferation markers were unravelled in the present investigation. Several pieces of evidence have demonstrated that ROS-induced oxidative damage is a major factor for the initiation of apoptotic cell death [46]. The present study showed that the decreased cellular antioxidants (SOD and GSH) and increased LPO in testes of ACR exposed rats were accompanied by increased testicular apoptosis. As downstream events of the apoptotic cascade, increased expression of the apoptosis-related protein, Caspase-3, was upregulated in the testes of the ACR-treated group compared with the control group (Fig. 6). During the apoptotic process, activation of caspase-3 is responsible for key protein cleavage and subsequent final cell disassembly [47]. On the other hand, PCNA, is considered a valuable cellular proliferation asset. It is an intra-nuclear polypeptide and a cofactor of DNA polymerase delta that is vital for replication and repair [48]. As spermatogenesis is a complex cell cycle of actively proliferating cells resulting in the formation of sperms, PCNA marker has been used to characterize spermatogonia and primary spermatocytes in all stages of the seminiferous tubules to evaluate spermatogenesis process [49]. ACR decreases the germ cell proliferation in rat testes as indicated by the drop of PCNA expression (Fig. 5). This diminished expression of PCNA by ACR has been previously documented [39]. ACR-associated apoptosis together with the decreased cell proliferation could be responsible for testicular dysfunction and might be the reason for the deteriorated sperm viability and mobility [45]. The significant elevation in PCNA expression in POS treated groups may be associated with the rise in cell cycle progression and the reduction of apoptosis. Furthermore, the antioxidant property of POS could prevent the initiation of apoptotic cell death and promote the cell cycle progression.

Testosterone, biosynthesized by Leydig cells, is necessary for sperm production and normal male reproductive function [50]. This steroidogenic pathway begins with the binding of the pituitary LH hormone to its receptor in the Leydig cells to initiate a series of signalling cascades. In detail, this pathway comprises the channelling and mobilization of cholesterol to the inner mitochondrial membrane by the steroidogenic acute regulatory protein, followed by the cholesterol side-chain cleavage by CYP11A1 enzyme (encoded by CYP11A1) forming pregnenolone, and conversion of pregnenolone to testosterone by the functions of steroidogenic enzymes series. These include 3β-hydroxysteroid dehydrogenase 1, cytochrome P450 17α-hydroxylase/17,20-lyase, and 17β-hydroxysteroid dehydrogenase 3 which is encoded by 17β3-HSD [51].

Serum testosterone level showed a significant reduction in ACR-exposed rats compared to control (Fig. 2). These results come in line with previous studies in rats [52, 53], weaning male rats [36], and mice [44]. It has been documented that the ACR-induced adverse effects on serum testosterone may be due to either the direct damaging effect exerted by ACR on Leydig cells, disruption of the testicular steroidogenesis, and/or stimulation of hepatic androgen biotransformation into metabolic products of low androgen receptor binding activity [10, 52, 54].

Both histopathological and immune-histochemical findings (Figs. 3, 5 & 6) confirmed the Leydig cells damage by the chronic ACR administration over 2 months which resulted in the marked reduction of serum testosterone and subsequently hypospermatogenesis [36, 44]. Furthermore, this study revealed that the mRNA expression levels of the two key steroidogenic enzymes CYP11A1 and 17β3-HSD were significantly down-regulated in the ACR-challenged rats (Fig. 7), which may also contribute to the decline of serum testosterone levels (Fig. 2). This was consistent with a previous study [10].

The present study highlighted the protective effects of the POS extract in the alleviation of ACR-induced male reproductive toxicity. Based on the findings obtained, concurrent administration of POS (200 and 400 mg/kg) hydro-alcoholic extract with chronic ACR exposure was efficient to protect rats against testicular dysfunction. POS attenuated the aforementioned ACR-induced reproductive ailments. The effectiveness of POS in improving spermatogenesis was evidenced by restoring the epididymal relative weight, sperm quality and quantity (Fig. 1).

Moreover, POS treatment significantly enhanced the ACR-disrupted antioxidant status as shown by the reduced testicular LPO and increased testicular SOD and GSH, which was noticed in literature upon receiving other treatments [43, 53, 55, 56]. In the same context, the POS treatment was effective to regulate apoptosis and cell proliferation in the testes by decreasing caspase 3 and increasing PCNA protein expression (Fig. 4, 5 and 6). These findings were notably supported by the ameliorative effects of POS on histologic testicular lesions compared with the ACR group (Fig. 3 and 4a). POS has been shown previously to impact the serum testosterone level (20) while our study outlined its potential effect counteracting the ACR-diminishing effect on the testosterone level. Furthermore, POS extract effectively reversed the ACR-downregulated steroidogenic CYP11A1 and 17β3-HSD (Fig. 7) which represents a new mechanism for POS protective effects on male fertility.

The ACR model exhibited a good clustering away from the negative control in different supervised and unsupervised multivariate data analyses confirming the model establishment. Interestingly, the co-treatment with 400 mg/kg POS showed a close clustering with the negative control indicating its potentiality to reverse the ACR-induced toxicities and the therapeutic potential of POS could be anticipated in a dose-dependent manner. Notably, the sperm motility and viability percentage showed the top-ranked VIP scores in PLS-DA analysis where relative 17β3-HSD mRNA expression followed by the percentage sperm count and mobility were top-ranked as discriminatory features in Random Forest analysis (Fig. 10).

These beneficial effects may be attributed to its arsenal of metabolites belonging to various chemical classes including a high content of polyunsaturated fatty acid and phytosterols [57]. For instance, ω3 fatty acids in purslane are responsible in restoring male fertility (13). In addition to, gallotannins, kaempferol, quercetin, apigenin and catecholamine content of POS [58, 59]. Consistently, our previous untargeted metabolomic analysis for the used POS in this study enabled the putative identification of 81 compounds in negative ionization mode and their structures were allocated as 48 fatty acyl/lipids, 11 flavonoids and its derivatives, 7 carbohydrates, 2 glycosylated hydroxy-cinnamic acid derivatives and miscellaneous terpenoids, steroids, lignan and purine nucleoside. However, fatty acyls, lipids and flavonoids were identified as the major three classes of metabolites in purslane seed [25].

‘Overall, our investigation conclusive proved the reproductive protection exerted by POS against ACR-induced infertility in rats by its antioxidant, antiapoptotic and steroidogenesis modulatory effects. However, there are some limitations in the current study. First, the number of animals used is low, however the difference between the single animals was not high as indicated by SD values with confirmatory validity provided by the meta- analysis of data. Second, our experiment did not adopt different dose gradients or time courses of POS treatment. In addition, how POS regulates the expression of steroidogenic enzymes also needs further study.’

## Conclusion

This report is the first to demonstrate the potential protective effects and the ameliorative mechanisms of *Portulaca oleracea* L*.* seed extract against ACR-induced testicular dysfunction. POS extract efficaciously prevented the development of ACR-induced reproductive toxicity in male rats escorted by the antioxidant and antiapoptotic-mediated fertility improvement. Moreover, the steroidogenic enzymes normalisation (CYP11A1 and 17β3-HSD) preserved the serum testosterone level that in turn maintained normal testicular function. Consequently, supplementation with POS extract may provide a potential therapeutic approach for testicular dysfunction resulting from acrylamide toxicity.

## Supplementary Information

**Additional file 1: Figure S1.** Dendogram of the hierarchical cluster analysis of the different experimental rat groups while exploring the effect of *Portulaca oleracea* seed extract (POS) on acrylamide (ACR) intoxicated rat model. **Figure S2.** Pearson r correlation heat-map of the biomarkers explored in the study of *Portulaca oleracea* seed extract effects on acrylamide intoxicated rats. 17β3-HSD; hydroxysteroid 17-beta dehydrogenase 3, CYP11A1; cytochrome P450, family 11 subfamily a polypeptide 1, GSH; Glutathione, MDA; Malondialdehyde, N_spermatozoa; normal spermatozoa percentage, PCNA; Caspase-3 and proliferating cell nuclear antigen, SOD; superoxide dismutase wt; weight.

## Data Availability

The dataset(s) supporting the conclusions of this article is (are) included within the article (and its additional file(s)).

## Abbreviations

17β3-HSD: Hydroxysteroid 17-beta dehydrogenase 3
ACR: Acrylamide
CYP11A1: Cytochrome P450, family 11, subfamily a, polypeptide 1
EPA: Environmental Protection Agency
FDA: Food and Drug Administration
GSH: Glutathione
LPO: Lipid peroxidation
MDA: Malondialdehyde
PCNA: Proliferating cell nuclear antigen
POS: *Portulaca oleracea* seed
SOD: Superoxide dismutase

## References

[1] Friedman M (2003). Chemistry, biochemistry, and safety of acrylamide. A review. J Agric Food Chem.

[2] Friedman M (2015). Acrylamide: inhibition of formation in processed food and mitigation of toxicity in cells, animals, and humans. Food Funct.

[3] Yilmaz B, Yildizbayrak N, Aydin Y, Erkan M (2017). Evidence of acrylamide-and glycidamide-induced oxidative stress and apoptosis in Leydig and Sertoli cells. Hum Exp Toxicol.

[4] Sun J, Li M, Zou F, Bai S, Jiang X, Tian L, et al. Protection of cyanidin-3-O-glucoside against acrylamide-and glycidamide-induced reproductive toxicity in leydig cells. Food Chem Toxicol. 2018;119:268–74. 10.1016/j.fct.2018.03.027. 10.1016/j.fct.2018.03.027 29574012

[5] Dobrovolsky VN, Pacheco-Martinez MM, McDaniel LP, Pearce MG, Ding W (2016). In vivo genotoxicity assessment of acrylamide and glycidyl methacrylate. Food Chem Toxicol.

[6] Ehlers A, Lenze D, Broll H, Zagon J, Hummel M, Lampen A (2013). Dose dependent molecular effects of acrylamide and glycidamide in human cancer cell lines and human primary hepatocytes. Toxicol Lett.

[7] Demetrio R, Casado M, Prats E, Faria M, Francesc P-C, Pérez Y, et al. Targeting redox metabolism: the perfect storm induced by acrylamide poisoning in the brain. Sci Rep. 2020;10:1. 10.1038/s41598-019-57142-y PMC6962170 31941973

[8] Matoso V, Bargi-Souza P, Ivanski F, Romano MA, Romano RM (2019). Acrylamide: a review about its toxic effects in the light of developmental origin of health and disease (DOHaD) concept. Food Chem.

[9] Khalil W, Ahmed H, Hanan F, Aly H, Eshak M (2014). Toxicological effects of acrylamide on testicular function and immune genes expression profile in rats. J Pharm Sci Rev Res.

[10] Yildizbayrak N, Erkan M (2018). Acrylamide disrupts the steroidogenic pathway in Leydig cells: possible mechanism of action. Toxicol Environ Chem.

[11] Wang E-T CD, Liu H, Yan H-Y, Yuan Y (2015). Protective effect of allicin against glycidamide-induced toxicity in male and female mice. Gen Physiol Biophys.

[12] Heydari M, Hashempur MH, Daneshfard B, Mosavat SH. Bioactive Foods as Dietary Intervention for Diabetes From the Perspective of Persian Medicine. In: Bioactive Food as Dietary Interventions for Diabetes: Academic press; 2019. p. 49–68.

[13] Abd El-Aziz H A. Sobhy MH, Ahmed KA, Abd El hameed AK, Rahman ZA, Hassan WA.Chemical and remedial effects of purslane (*Portulaca oleracea*) plant. Life Sci J. 2014;11(6):31–42.

[14] Londonkar R, Nayaka HB (2011). Phytochemical and antimicrobial activities of Portulaca oleracea L. J Pharm Res.

[15] Boroushaki MT, Boskabady MH, Malek F (2010). Antitussive effect of *Portulaca oleracea* L. in Guinea pigs. Iranian J Pharm Res.

[16] Chan K, Islam MW, Kamil M (2000). al. E: analgesic and anti-inflammatory effects of Portulaca oleracea L. subsp. sativa (haw.) Celak. J Ethnopharmacol.

[17] Bai Y, Zang X, Ma J, Xu G (2016). Anti-diabetic effect of *portulaca oleracea* l. polysaccharideandits mechanism in diabetic rats. Int J Mol Sci.

[18] Nayaka HB, Londonkar RL, Andumesh MK (2014). Evaluation Of *Portulaca oleracea* L. For Anti-Fertility Effect In Female Albino Rats. Int J Pharm Pharm Sci.

[19] Ahangarpour A, Lamoochi Z, Fathi Moghaddam H, Mansouri SM (2016). Effects of Portulaca oleracea ethanolic extract on reproductive system of aging female mice. Int J Reprod Biomed.

[20] Lev E, Amar Z (2002). Ethnopharmacological survey of traditional drugs sold in the kingdom of Jordan. J Ethnopharmacol.

[21] Abbas MA (2017). Is the use of plants in Jordanian folk medicine for the treatment of male sexual dysfunction scientifically based? Review of in vitro and in vivo human and animal studies. Andrologia.

[22] Al-Bishri WM, Abdel-Reheim ES, Zaki AR (2017). Purslane protects against the reproductive toxicity of carbamazepine treatment in pilocarpine-induced epilepsy model. Asian Pac J Trop Biomed.

[23] Pharmacopoeia C (2010). Pharmacopoeia of the PR China. Press Chem Ind Beijing.

[24] Safaeian L, Baniahmad B, Esfandiari Z, Alavi SA (2017). Portulaca oleracea seeds extract does not prevent dexamethasone-induced hypertension in rats. J Herbmed Pharmacol.

[25] Farag OM, Abd-Elsalam RM, Ogaly HA, Ali SE, El Badawy SA, Alsherbiny MA, et al. Metabolomic profiling and Neuroprotective effects of Purslane seeds extract against acrylamide toxicity in Rat’s brain. Neurochem Res. 2021;46(4):819–42. 10.1007/s11064-020-03209-6. 10.1007/s11064-020-03209-6 33439429

[26] Wang H, Huang P, Lie T, Li J, Hutz RJ, Li K, et al. Reproductive toxicity of acrylamide-treated male rats. Reprod Toxicol. 2010;29(2):225–30. 10.1016/j.reprotox.2009.11.002. 10.1016/j.reprotox.2009.11.002 19903525

[27] Oliveira PF, Tomas GD, Dias TR, Martins AD, Rato L, Alves MG, et al. White tea consumption restores sperm quality in prediabetic rats preventing testicular oxidative damage. Reprod BioMed Online. 2015;31(4):544–56. 10.1016/j.rbmo.2015.06.021. 10.1016/j.rbmo.2015.06.021 26276042

[28] Rato L, Alves M, Dias T, Lopes G, Cavaco J, Socorro S, et al. High-energy diets may induce a pre-diabetic state altering testicular glycolytic metabolic profile and male reproductive parameters. Andrology. 2013;1(3):495–504. 10.1111/j.2047-2927.2013.00071.x. 10.1111/j.2047-2927.2013.00071.x 23495257

[29] Kalaivani M, Saleena UV, Katapadi KGK, Kumar YP, Nayak D (2018). Effect of acrylamide ingestion on reproductive organs of adult male wistar rats. J Clin Diagn Res.

[30] Ohkawa H, Ohishi N, Yagi K (1979). Assay for lipid peroxides in animal tissues by thiobarbituric acid reaction. Anal Biochem.

[31] Bancroft JD, Gamble M (2008). Theory and practice of histological techniques: Elsevier health sciences.

[32] Johnsen SG (1970). Testicular biopsy score count–a method for registration of spermatogenesis in human testes: normal values and results in 335 hypogonadal males. Hormone Res Paediatr.

[33] Soliman GA, Saeedan AS, Abdel-Rahman RF, Ogaly HA, Abd-Elsalam RM, Abdel-Kader MS (2019). Olive leaves extract attenuates type II diabetes mellitus-induced testicular damage in rats: molecular and biochemical study. Saudi Pharm J.

[34] Abdel-Rahman RF, Soliman GA, Saeedan AS, Ogaly HA, Abd-Elsalam RM, Alqasoumi SI, et al. Molecular and biochemical monitoring of the possible herb-drug interaction between Momordica charantia extract and glibenclamide in diabetic rats. Saudi Pharm J. 2019;27(6):803–16. 10.1016/j.jsps.2019.05.002. 10.1016/j.jsps.2019.05.002 PMC6733788 31516323

[35] Chong J, Soufan O, Li C, Caraus I, Li S, Bourque G, et al. MetaboAnalyst 4.0: towards more transparent and integrative metabolomics analysis. Nucleic Acids Res. 2018;46(W1):W486–94. 10.1093/nar/gky310. 10.1093/nar/gky310 PMC6030889 29762782

[36] Pourentezari M, Talebi A, Abbasi A, Khalili MA, Mangoli E, Anvari M (2014). Effects of acrylamide on sperm parameters, chromatin quality, and the level of blood testosterone in mice. Iran J Reprod Med.

[37] Christina OU, Daniel UO (2015). Effects of acrylamide on the reproductive hormones and sperm quality in male rats. Int J Sci Res.

[38] Ma Y, Shi J, Zheng M, Liu J, Tian S, He X, et al. Toxicological effects of acrylamide on the reproductive system of weaning male rats. Toxicol Ind Health. 2011;27(7):617–27. 10.1177/0748233710394235. 10.1177/0748233710394235 21415092

[39] Kacar S, Sahinturk V, Can B, Musmul A (2018). L-cysteine partially protects against acrylamide-induced testicular toxicity. Balkan Med J.

[40] Prathima P, Venkaiah K, Pavani R, Daveedu T, Munikumar M, Gobinath M, et al. Sainath SB: alpha-lipoic acid inhibits oxidative stress in testis and attenuates testicular toxicity in rats exposed to carbimazole during embryonic period. Toxicol Rep. 2017;4:373–81. 10.1016/j.toxrep.2017.06.009. 10.1016/j.toxrep.2017.06.009 PMC5615143 28959662

[41] Baba SP, Bhatnagar A (2018). Role of Thiols in oxidative stress. Curr Opin Toxicol.

[42] Singhal SS, Singh SP, Singhal P, Horne D, Singhal J, Awasthi S (2015). Antioxidant role of glutathione S-transferases: 4-Hydroxynonenal, a key molecule in stress-mediated signaling. Toxicol Appl Pharmacol.

[43] He Y, Tan D, Mi Y, Bai B, Jiang D, Zhou X, et al. Effect of epigallocatechin-3-gallate on acrylamide-induced oxidative stress and apoptosis in PC12 cells. Hum Exp Toxicol. 2017;36(10):1087–99. 10.1177/0960327116681648. 10.1177/0960327116681648 27920337

[44] Yang HJ, Lee SH, Jin Y, Choi JH, Han CH, Lee MH (2005). Genotoxicity and toxicological effects of acrylamide on reproductive system in male rats. J Vet Sci.

[45] Jeremy M, Gurusubramanian G, Roy VK (2019). Vitamin D3 regulates apoptosis and proliferation in the testis of D-galactose-induced aged rat model. Sci Rep.

[46] Tabeshpour J, Mehri S, Abnous K, Hosseinzadeh H (2019). Neuroprotective effects of thymoquinone in acrylamide-induced peripheral nervous system toxicity through MAPKinase and apoptosis pathways in rat. Neurochem Res.

[47] Pan X, Yan D, Wang D, Wu X, Zhao W, Lu Q, et al. Mitochondrion-mediated apoptosis induced by acrylamide is regulated by a balance between Nrf2 antioxidant and MAPK signaling pathways in PC12 cells. Mol Neurobiol. 2017;54(6):4781–94. 10.1007/s12035-016-0021-1. 10.1007/s12035-016-0021-1 27501804

[48] Kanter M, Aktas C, Erboga M (2013). Curcumin attenuates testicular damage, apoptotic germ cell death, and oxidative stress in streptozotocin-induced diabetic rats. Mol Nutr Food Res.

[49] Kang MJ, Kim MK, Terhune A, Park JK, Kim YH, Koh GY (1997). Cytoplasmic localization of cyclin D3 in seminiferous tubules during testicular development. Exp Cell Res.

[50] O'Donnell L, Stanton P, de Kretser DM, Feingold KR, Anawalt B, Boyce A, Chrousos G, de Herder WW, Dungan K, Grossman A, Hershman JM, Hofland J, Kaltsas G (2000). Endocrinology of the male reproductive system and spermatogenesis. Endotext.

[51] Chen H, Ge RS, Zirkin BR (2009). Leydig cells: from stem cells to aging. Mol Cell Endocrinol.

[52] Yassa HA, George SM, Refaiy Ael R, Moneim EM (2014). Camellia sinensis (green tea) extract attenuate acrylamide induced testicular damage in albino rats. Environ Toxicol.

[53] Lebda M, Gad S, Gaafar H (2014). Effects of lipoic acid on acrylamide induced testicular damage. Mater Soc.

[54] Omar HE-DM (2015). Acrylamide induced testicular toxicity in rats: protective effect of garlic oil. Biomarkers.

[55] Yousef MI, El-Demerdash FM (2006). Acrylamide-induced oxidative stress and biochemical perturbations in rats. Toxicology.

[56] ALTURFAN EI, Beceren A, ŞEHİRLİ AÖ, Demiralp ZE, ŞENER G, OMURTAG GZ (2012). Protective effect of N-acetyl-L-cysteine against acrylamide-induced oxidative stress in rats. Turk J Vet Anim Sci.

[57] Dehghan F, Soori R, Gholami K, Abolmaesoomi M, Yusof A, Muniandy S, et al. Purslane (Portulaca oleracea) seed consumption and aerobic training improves biomarkers associated with atherosclerosis in women with type 2 diabetes (T2D). Sci Rep. 2016;6(1):37819. 10.1038/srep37819. 10.1038/srep37819 PMC5137030 27917862

[58] Zhou YX, Xin HL, Rahman K, Wang SJ, Peng C, Zhang H (2015). *Portulaca oleracea* L.: a review of phytochemistry and pharmacological effects. Biomed Res Int.

[59] Zhu H, Wang Y, Liu Y, Xia Y, Tang T (2010). Analysis of flavonoids in Portulaca oleracea L. by UV–vis spectrophotometry with comparative study on different extraction technologies. Food Anal Methods.

[60] Perciedu Sert N, Ahluwalia A, Alam S, Avey MT, Baker M, Browne WJ, et al. Reporting animal research: Explanation and elaboration for the ARRIVE guidelines 2.0. PLoS Biol. 2020;18(7):e3000411. 10.1371/journal.pbio.3000411 PMC7360025 32663221

