# Supplementary material for: Portulaca oleracea seeds’ extract alleviates acrylamide-induced testicular dysfunction by promoting oxidative status and steroidogenic pathway in rats
Source: BMC Complement Med Ther. 2021 Apr 14;21:122. doi: 10.1186/s12906-021-03286-2 (PMC8045344; doi:10.1186/s12906-021-03286-2)
Supplement: Supplementary file 1 — Additional file 1: Figure S1. Dendogram of the hierarchical cluster analysis of the different experimental rat groups while exploring the effect of Portulaca oleracea seed extract (POS) on acrylamide (ACR) intoxicated rat model. Figure S2. Pearson r correlation heat-map of the biomarkers explored in the study of Portulaca oleracea seed extract effects on acrylamide intoxicated rats. 17β3-HSD; hydroxysteroid 17-beta dehydrogenase 3, CYP11A1; cytochrome P450, family 11 subfamily a polypeptide 1, GSH; Glutathione, MDA; Malondialdehyde, N_spermatozoa; normal spermatozoa percentage, PCNA; Caspase-3 and proliferating cell nuclear antigen, SOD; superoxide dismutase wt; weight. [file 12906_2021_3286_MOESM1_ESM.docx]

***Portulaca oleracea* seeds’ extract alleviates acrylamide-induced testicular dysfunction by promoting oxidative status and steroidogenic pathway in rats**

**Ola M. Farag^a^, Reham M. Abd-Elsalam^b^, , Shymaa A. El Badawy^c^, Hanan A. Ogaly^d,e^, Muhammad A. Alsherbiny^f,g^ and Kawkab A. Ahmed^b*^**

^a^ General Organization for Veterinary Services.

^b^ Department of Pathology, Faculty of Veterinary Medicine, Cairo University, Giza 12211, Egypt.

^c^ Department of Pharmacology, Faculty of Veterinary Medicine, Cairo University, Giza 12211, Egypt.

^d^ Department of Chemistry, College of Science, King Khalid University, Abha, Saudi Arabia.

^e^ Department of Biochemistry, Faculty of Veterinary Medicine, Cairo University, Giza 12211, Egypt.

^f^ Department of Pharmacognosy, Faculty of Pharmacy, Cairo University, Cairo 12613, Egypt

^g^ NICM Health Research Institute, Western Sydney University, Westmead, NSW 2145, Australia

* For correspondence


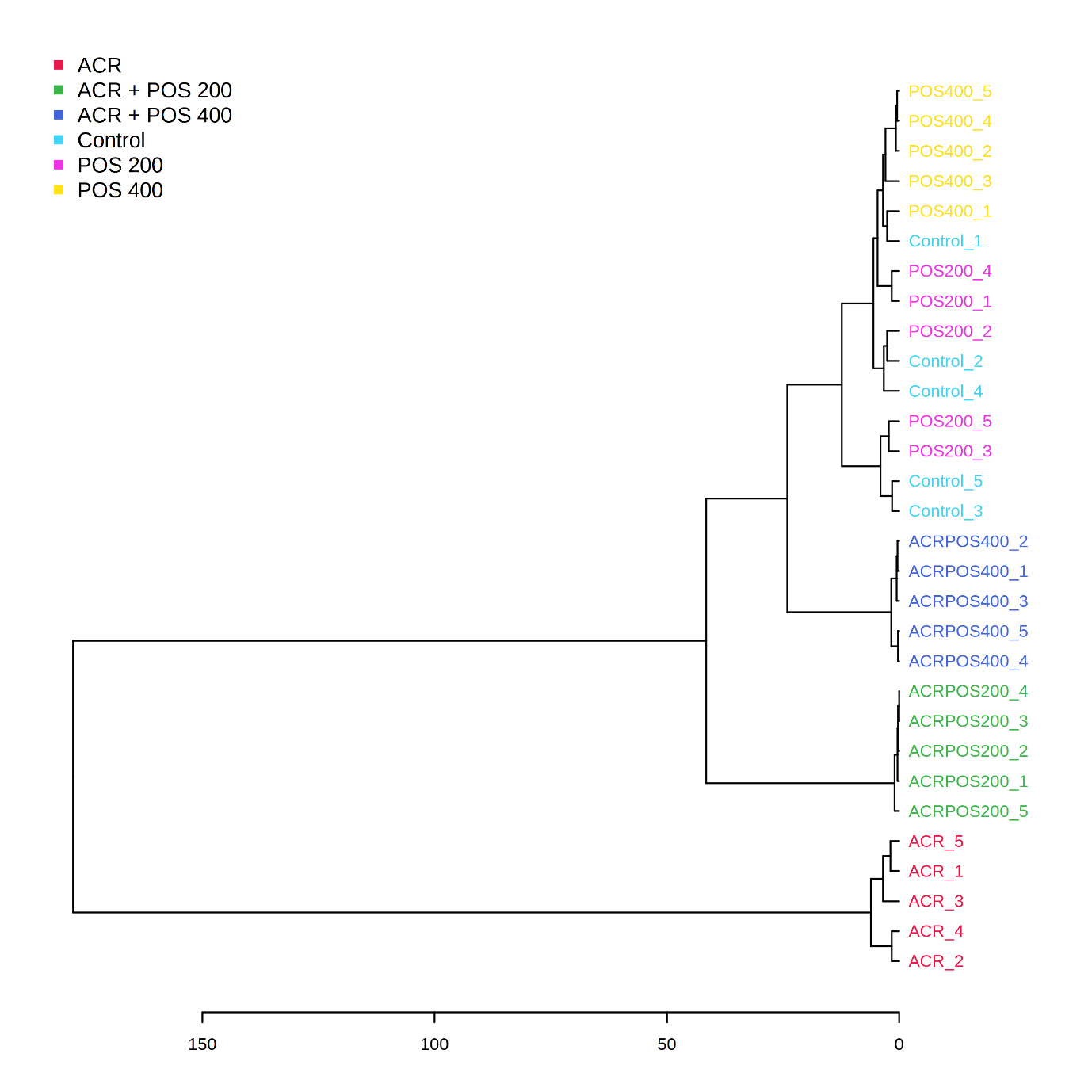


Figure S1 Dendogram of the hierarchical cluster analysis of the different experimental rat groups while exploring the effect of *Portulaca oleracea* seed extract (POS) on acrylamide (ACR) intoxicated rat model.


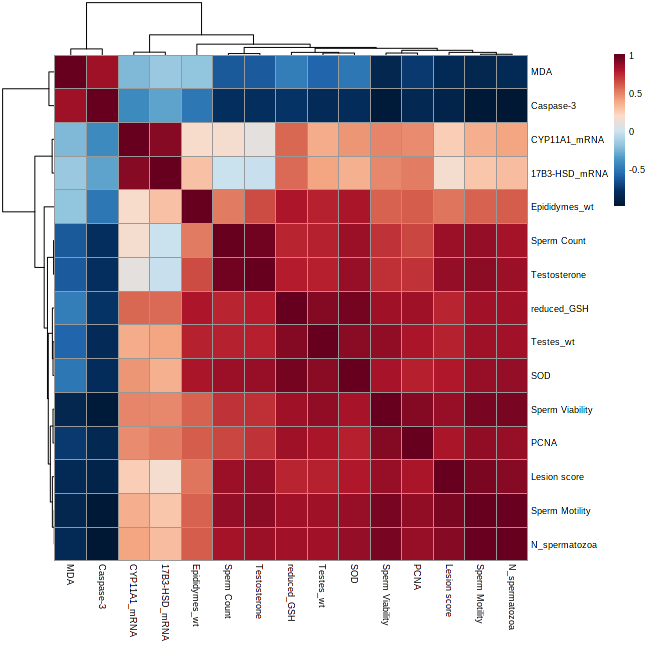


Figure S2 Pearson r correlation heat-map of the biomarkers explored in the study of *Portulaca oleracea* seed extract effects on acrylamide intoxicated rats. 17β3-HSD; hydroxysteroid 17-beta dehydrogenase 3, CYP11A1; cytochrome P450, family 11 subfamily a polypeptide 1, GSH; Glutathione, MDA; Malondialdehyde, N_spermatozoa; normal spermatozoa percentage, PCNA; Caspase-3 and proliferating cell nuclear antigen, SOD; superoxide dismutase wt; weight
